# Drivers of diversification in sharks and rays (Chondrichthyes: Elasmobranchii)

**DOI:** 10.3389/fevo.2024.1530326

**Published:** 2025-01-10

**Authors:** Joel H. Gayford, Patrick L. Jambura

**Affiliations:** 1 College of Science and Engineering, James Cook University, Townsville, Australia; 2 Shark Measurements, London, United Kingdom; 3 Department of Palaeontology, Faculty of Earth Sciences, Geography and Astronomy, University of Vienna, Vienna, Austria

**Keywords:** biotic interactions, trait evolution, macroevolution, speciation, extinction, ecological opportunity

## Abstract

Elasmobranchs (sharks and rays) are a charismatic lineage of unquestionable ecological importance in past and present marine ecosystems. Represented by over 1200 species, elasmobranchs have undergone substantial shifts in taxonomic diversity since their origin. Quantifying these diversification trends and their underlying causes improves our understanding of macroevolutionary processes and the factors influencing community composition through deep time. Studies addressing drivers of diversification in Elasmobranchii have yielded conflicting results; some report clear relationships between specific traits and diversification events, whilst others fail to find support for such relationships. There is also some evidence to suggest that biotic interactions or environmental factors (global climatic change and tectonic events) have shaped elasmobranch diversification dynamics. In this review, we summarise the diversification dynamics of elasmobranchs over their evolutionary history, before considering the evidence for the three principal hypothesised drivers of diversification in this clade: trait evolution, biotic interactions, and environmental change. Finally, we discuss major limitations in the field, and how discordant methodologies and data sources hamper our current understanding of diversification in Elasmobranchii. Whilst future studies will undoubtedly be required to further unravel this complex relationship, no single factor can be considered the sole satisfactory explanation for observed deep time diversification trends in Elasmobranchii to the exclusion of the other.

## 1 Introduction

Diversification dynamics refer to spatiotemporal variation in rates of speciation and extinction in a given clade or set of lineages (Aguilée et al., 2018) and consequently define the accumulation and demise of biological diversity through time. Speciation and extinction rates are influenced by various biotic and abiotic factors (Helmstetter et al., 2023; Lewitus and Morlon, 2018), and disentangling their relative importance through deep time is important as it shapes our understanding of adaptation, ecological interactions and community composition in past ecosystems, as well as our understanding of mass extinction events.

Elasmobranchii (sharks and rays) is a speciose clade that has undergone radical changes in diversity over the past 400 million years (Heinicke et al., 2009). Elasmobranchs exhibit variation in morphology and body size, ecology, physiology, reproductive biology and life history (Ebert et al., 2021; Last et al., 2016) – all traits that could feasibly influence diversification dynamics in this clade. Current understanding of diversification trends in elasmobranchs is largely based on the fossil record (Kriwet et al., 2009; Maisey et al., 2004; Underwood, 2006), however recently phylogenetic approaches have been employed to study potential drivers of diversification (Brée et al., 2022; Marion et al., 2024; Sorenson et al., 2014). Interestingly, these studies reach conflicting conclusions regarding which factors have been more important in shaping elasmobranch diversification trends (Marion et al., 2024). Given the dire conservation status of many elasmobranch species (Dulvy et al., 2021), improving our understanding of diversification drivers in this clade may provide valuable context through which we can assess vulnerability to extinction in contemporary lineages.

In this review, we discuss diversification trends in Elasmobranchii and their hypothesized drivers in an attempt to resolve the apparent conflicting findings of recent studies.

## 2 Diversification/turnover trends in elasmobranchs

**Figure 1:**
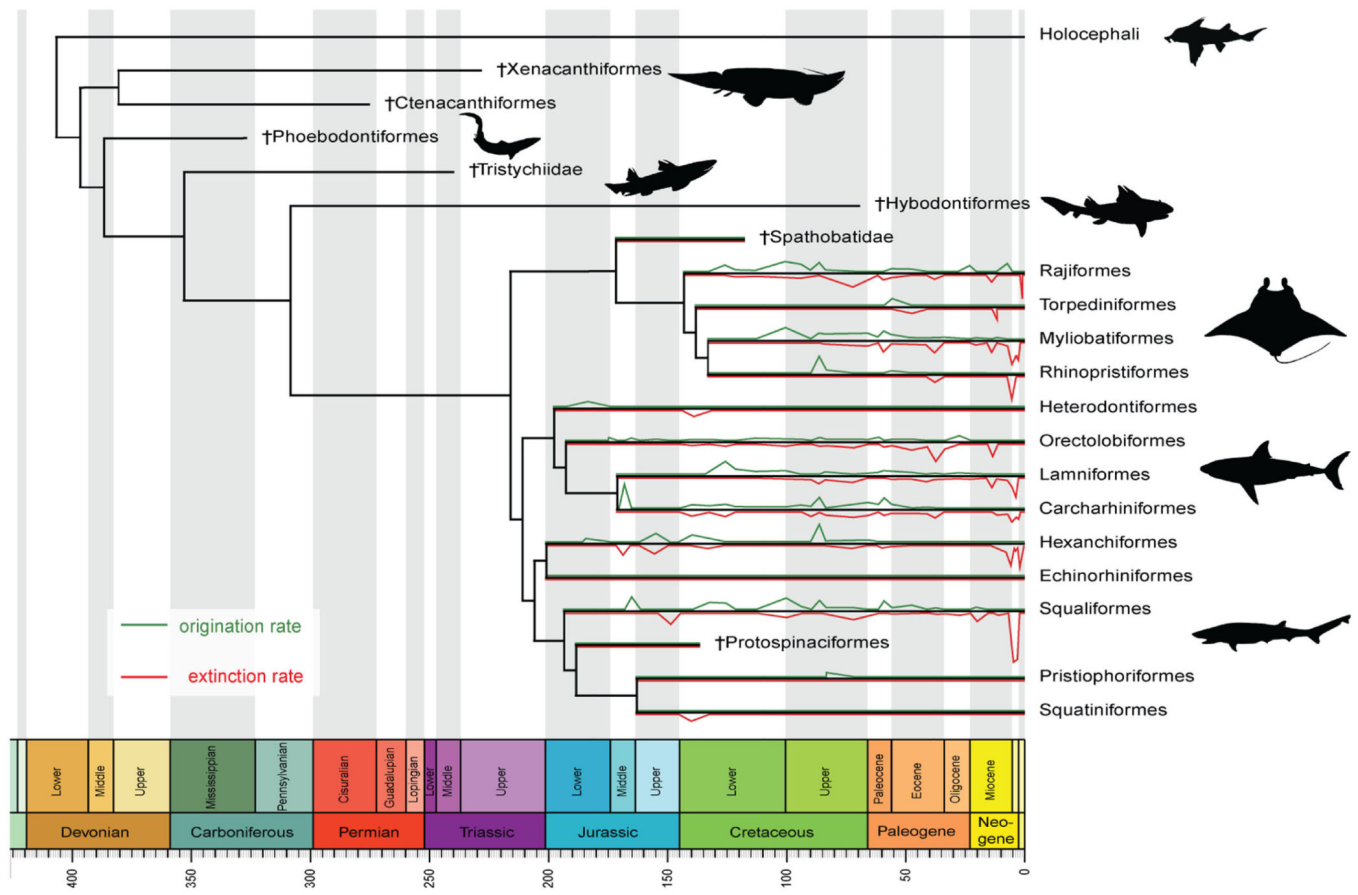
Diversification dynamics in crown-group elasmobranchs (i.e., Neoselachii). The topology of the phylogenetic tree is based on Frey et al., 2019 and Jambura et al., 2023, and time calibration was performed in the R package paleotree (Bapst, 2012; R Core Team, 2024) using the a-posteriori “minimum branch length” (MBL) dating method. Minimum ages for each branch followed Cappetta (2012). Rates of origination and extinction were extracted from the Paleobiology Database (PBDB; Peters and McClennen, 2016).

Elasmobranchs originated during the Devonian (419-358.9 Ma) (Figure 1; Grogan et al., 2012), potentially radiating into niches vacated by the extinction of stem gnathostomes during the Late Devonian Hangenberg event (Sallan and Coates, 2010). Molecular phylogenies suggest the divergence between sharks and rays occurred during this time (Sorenson et al., 2014; Heinicke et al., 2009), although conclusive fossil evidence of Paleozoic crown group elasmobranchs (i.e., Neoselachii) has yet to be found, with the fossil record indicating that this split did not occur before the Early Jurassic (Stumpf and Kriwet, 2019). During the Paleozoic, elasmobranchs were dwarfed by Holocephali and osteichthyans in terms of diversity (Grogan et al., 2012; Schnetz et al., 2024; Whitenack et al., 2022). The end of the Paleozoic area is marked by dramatic declines in chondrichthyan (and presumably elasmobranch) diversity during the ‘great dying’ Permo-Triassic mass extinction event (Schnetz et al., 2024) that decimated over 90% of marine life (Huang et al., 2023).

Most living elasmobranch families originated during the Mesozoic (Heinicke et al., 2009; Maisey, 2012). Phylogenetic and fossil evidence suggest a rapid period of Early Jurassic neoselachian diversification in which many extant lineages (e.g., Hexanchiformes, Heterodontiformes, and Orectolobiformes) first originated (Kriwet et al., 2009; Underwood, 2006). The Bathonian (Middle Jurassic) represents another key stage in elasmobranch diversification with several now-speciose groups radiating, and Lamniformes appearing for the first time (Figure 1; Jambura et al., 2019). The Late Jurassic is typified by stasis, with low diversification rates thought to be associated with a lack of global-scale biotic or climatic shifts in marine ecosystems (Guinot and Cavin, 2016; Kriwet and Klug, 2008; Kriwet et al., 2009). Although Early Cretaceous elasmobranchs are poorly understood, most neoselachian orders (except Torpediniformes) were likely present, with the Albian (~113-100.5 Ma) showing significant ecological diversification, particularly in Lamniformes, Squaliformes, and Batoidea (Underwood, 2006; Underwood et al., 1999; Kriwet et al., 2009; Maisey, 2012). The Late Cretaceous appears to feature a steady increase in diversity of neritic elasmobranchs (Underwood, 2006) prior to the Cretaceous-Paleogene mass extinction in which declines surpassed 60% (Kriwet and Benton, 2004; Guinot and Condamine, 2023). Recovery was uneven, and pre-extinction levels of diversity were not reached until after the Paleocene (Guinot and Condamine, 2023).

## 3 Drivers of diversification: trait evolution

Several studies have identified trends between elasmobranch diversification dynamics and the evolution of specific ecological and morphological traits (Table 1). Rates of diversification in sharks mirror rates of mandible evolution and dentition, particularly among Lamniformes and Carcharhiniformes (Bazzi et al., 2021; López-Romero et al., 2023). Consequently, the evolution of traits relating to prey handling/acquisition may have facilitated radiation into new trophic niches (Bazzi et al., 2021). Moreover, loss of sperm storage potential in females is associated with elevated extinction rates, potentially explaining the depauperate nature of Lamniformes and Rhinopristiformes (Lamarca et al., 2024).

Fossil evidence also indicates that trait evolution has played an important role in elasmobranch diversification trends: it is thought that the rapid radiation of several clades following the end-Triassic mass extinction was facilitated by life-history traits including small body size and oviparity, enabling rapid adaptation to novel ecological conditions (Kriwet et al., 2009). Intriguingly, this conflicts with a subsequent study addressing diversification trends over a greater temporal scale, which found that elevated diversification rates were associated with viviparity and increases in body size (Mull et al., 2024). There are several cases in which the evolution of unique traits appears to be associated with changes in diversification dynamics within specific elasmobranch clades. One example is the evolution of bioluminescent lateral photophores in etmopterid sharks (Duchatelet et al., 2021; Ferrón, 2023). These markings are associated with elevated speciation rates and may increase the probability of reproductive isolation building up between populations (Claes et al., 2015). This likely explains the unusually speciose nature of Etmopteridae given the clade’s age, a trend observed in many lineages with bioluminescent markings (Davis et al., 2014; Ellis and Oakley, 2016). A further example is found in lamniform sharks, where speciation and extinction rates were found to be negatively correlated with tooth size (Condamine et al., 2019). Thus, in at least some elasmobranch lineages, there is evidence for the role of trait evolution in shaping diversification dynamics.

## 4 Drivers of diversification: environmental change

Not all studies have found clear relationships between trait evolution and diversification dynamics in Elasmobranchii. Marion et al. (2024) tested for associations between diversification rate and several traits (including body size and reproductive mode) in sharks and found no such associations. Moreover, several of the aforementioned studies, whilst finding support for relationships between diversification dynamics and trait evolution in some clades, fail to find evidence of such relationships in others (Condamine et al., 2019; Mull et al., 2024). Where direct evidence of trait-mediated diversification is absent, environmental perturbations, including tectonic events (Couvreur et al., 2021), eustatic sea level changes (Nardin and Lefebvre, 2010), and vicariance events (Poulakakis et al., 2012) is often cited as underlying diversification dynamics.

Consequently, periods of elevated elasmobranch diversification have been attributed to the exploitation of ecological opportunity (Brée et al., 2022; Corrigan and Beheregaray, 2009; Kriwet and Benton, 2004; Mull et al., 2024). Phylogenetic studies indicate that several Cenozoic instances of rapid elasmobranch diversification are linked to known biogeographic events. Continental fragmentation and eustatic sea level rises (and the associated diversification of coral reefs) during the Oligocene and Miocene likely facilitated bursts of speciation in Carcharhiniformes and Orectolobidae (Boyd and Seitz, 2021; Brée et al., 2022; Corrigan and Beheregaray, 2009; Mull et al., 2024; Sorenson et al., 2014; Wood, 1999). Other groups (e.g., Rajiformes and Scyliorhinidae) diversified into vacant deep-water niches upon the Eocene formation of multiple, deep oceanic passages (Long, 1994; Mull et al., 2024). More broadly, a combination of environmental factors including palaeotemperature, eustatic sea level, continental fragmentation and ocean circulation, prey availability, and productivity appear to explain diversification trends of elasmobranchs across the Paleozoic, Mesozoic, and Cenozoic (Bazzi et al., 2018; Guinot and Cavin, 2016; Villafaña et al., 2019).

Extinction rate can also be modulated by environmental conditions, notably during mass extinctions - major changes in abiotic and or biotic conditions result in the raising of extinction levels to far above background levels (Jablonski, 2005). For example, the K-Pg event that extirpated over 60% of elasmobranch diversity (Kriwet and Benton, 2004; Guinot and Condamine, 2023) is thought to have been driven by an asteroid impact and associated effects on global-scale photosynthesis (Schulte et al., 2010). Mass extinctions are particularly relevant due to the scale of their impact. Previously abundant groups can be exterminated over short periods of time, as in the case of most early chondrichthyans, that were extirpated during the End-Devonian events (Whitenack et al., 2022). There is also evidence for environmentally mediated declines in shark lineages on smaller scales. Fossil data indicate that the demise of lamniform sharks over the past 20 million years likely occurred in part due to global cooling (Condamine et al., 2019). It could be argued that due to the sheer geographic and taxonomic scale of mass extinction events, environmental change is more important to elasmobranch diversification trends than trait evolution, as has been posited recently (Marion et al., 2024).

## 5 Drivers of diversification: biotic interactions

In addition to trait evolution and environmental change, biotic interactions—such as clade competition, replacement, and predator-prey dynamics—are a third hypothesized major driver of diversification (Maas et al., 1988; Rosenzweig and McCord, 1991).

There is some evidence for these mechanisms in elasmobranchs. Fossil-based diversification analyses indicate that post-Cretaceous reductions of lamniform diversity were in part due to clade competition with Carcharhiniformes, as lamniform speciation rates correlate with carcharhiniform diversity (Condamine et al., 2019). Moreover, negative diversity-dependent speciation rates among medium-sized lamniform sharks indicate that within-clade competition may have contributed to this decline (Condamine et al., 2019). Requiem and hammerhead sharks likely radiated into vacant ecological niches as lamniform taxa went extinct (Friedman and Sallan, 2012; Kriwet and Benton, 2004). However, there is a notable lack of studies in the literature testing for direct associations between elasmobranch taxonomic diversity and speciation/extinction rates, and at present Condamine et al. (2019) is the only exception. Hence, whilst clade competition has undoubtedly played some role in shaping the diversification trajectories of lamniform sharks, the extent to which this applies to other lineages remains uncertain.

There is also some evidence for the role of predator-prey dynamics in shaping elasmobranch diversity trends. Patterns of dental disparity and morphological turnover across the K-Pg mass extinction indicate that biotic interactions relating to prey availability (and ensuing trophic cascades) may have initiated several Cenozoic shark radiations including multiple carcharhiniform diversification events (Bazzi et al., 2018).

## 6 Drivers of diversification: synthesis

Whether through ecological opportunity or mass extinction, environmental perturbations are often suggested to be the main driver of elasmobranch diversification trends (Condamine et al., 2019; Guinot and Condamine, 2023; Whitenack et al., 2022). However, as the interface between genotype and environment, the combination of traits possessed by a taxon is an important determinant of survival in the face of such perturbations (Cardillo et al., 2005; Jablonski, 2005; Orzechowski et al., 2015; Zamudio et al., 2016). Mass extinctions are typically selective, meaning that the extinction rate of a given lineage is linked to the traits possessed by its constituent taxa (Cardillo et al., 2005; Jablonski, 2005; Orzechowski et al., 2015). Considering events such as the Permo-Triassic extinction, driven by deoxygenation, acidification, and warming (Dal Corso et al., 2022), taxa with broader thermal tolerance would have been more likely to persist (Vázquez and Clapham, 2017). There is empirical evidence for the role of certain traits in determining the extinction selectivity in Elasmobranchii: The Cretaceous-Paleogene extinction event was particularly disastrous for durophagous and benthic elasmobranchs (Guinot and Condamine, 2023). Whilst geographic ranges and environmental tolerance rather than individual traits are generally thought to determine selectivity at higher taxonomic levels (Jablonski, 2005), the tolerance and geographic distribution of elasmobranchs at the clade level are themselves trait-dependent (Villafaña and Rivadeneira, 2018).

The exploitation of ecological opportunity is also fundamentally linked to trait evolution and the role of traits in biotic interactions. Ecological opportunity arises through geographical colonisation, extinction of antagonists, and the origin of key innovations (Stroud and Losos, 2016; Yoder et al., 2010). The latter is a form of trait evolution in itself, but both other sources of ecological opportunity depend critically on species’ traits and how they are used to interact with other organisms. Geographical colonization, as observed in the Oligocene/Miocene radiations of Carcharhiniformes and Orectolobiformes (Boyd and Seitz, 2021; Sorenson et al., 2014), requires that taxa possess or rapidly evolve the necessary traits to persist under novel environmental conditions (Yoder et al., 2010), and that they can coexist with or outcompete any lineages occupying similar niches in the new habitat. Whilst these diversification events have been linked to the proliferation of coral reefs and other biogeographical changes (Boyd and Seitz, 2021; Sorenson et al., 2014), trophic interactions and the morphological adaptations through which they manifest were also crucial to the persistence and radiation of these now speciose orders (Bazzi et al., 2021).

This is of particular relevance to the repeated invasions of benthic and pelagic environments that have occurred throughout elasmobranch phylogeny (Sorenson et al., 2014). In the case of Oligocene/Miocene radiations, whilst eustasy and continental fragmentation were important (Sorenson et al., 2014), these environmental changes would also have required incumbent taxa to maneuver structurally complex environments and novel interspecific interactions (Bellwood and Wainwright, 2002; Darling et al., 2017; Sorenson et al., 2014). Consequently, tectonic/climatic variation, ecomorphological specialization, and possibly biotic interactions, were all essential components of carcharhiniform and orectolobiform radiations in the Cenozoic (Bazzi et al., 2018, 2021; López-Romero et al., 2023; Sorenson et al., 2014). Indeed, analysis of the Neogene chondrichthyan fossil record suggests that biogeographic range shifts at various spatiotemporal scales were modulated by traits including body size and salinity/temperature preferences (Villafaña and Rivadeneira, 2018), with ensuing diversification events likely reliant on the interplay between biogeography and traits, and its consequences for the outcome of trophic interactions.

The ecological opportunity afforded by the extirpation of antagonists, and the ability of lineages to exploit it, is also dependent on trait evolution. Competition, both with groups such as stem gnathostomes (Sallan and Coates, 2010) and between different elasmobranch lineages (Condamine et al., 2019), is thought to have played an important role in driving elasmobranch diversification dynamics. However, the impact of competition and other biotic interactions on diversification events depends on both the extent to which species’ traits overlap, and spatiotemporal characteristics of the environment (Pastore et al., 2021). In the case of predator-prey dynamics, dentition prey handling/acquisition is intrinsically associated with morphological evolution and may have been critical to Cenozoic shark radiations (Bazzi et al., 2018). Moreover, following loss of antagonists, the ability of a lineage to persist and diversify into a vacated niche (and outcompete other lineages) will depend on the suite of traits possessed by the incumbent, and the rate at which novel traits can evolve (Yoder et al., 2010).

## 7 How much do we really know about drivers of diversification in Elasmobranchii?

Despite much interest in the evolutionary history of cartilaginous fishes, few studies have empirically assessed drivers of diversification dynamics in elasmobranchs, and those that do often reach conflicting conclusions (Table 1). One potential explanation is the methodological differences between studies predominantly drawing upon the fossil record (e.g., Kriwet et al., 2009), and those focussing on neonatological data (e.g., Mull et al., 2024). The former has obvious limitations such as preservation biases and time averaging (Behrensmeyer et al., 2000). However, fossil data are the only direct evidence of past taxonomic diversity, and the exclusion of fossil data in phylogenetic studies represents a major limitation (Quental and Marshall, 2010). Moreover, the uncertainty/error associated with ancestral state reconstruction approaches, frequently employed in such studies, increases the further back in time one looks (Royer-Carenzi and Didier, 2016). Additionally, conclusions derived from molecular clock approaches using fossil calibration are only as reliable as the underlying palaeontological data (Müller and Reisz, 2005; Parham and Irmis, 2008). Using calibration points with uncertain phylogenetic affiliations or ages can lead to discrepancies between molecular clock estimates and the fossil record, affecting our interpretations of the timing and drivers of diversification.

Taxon sampling and taxonomic level represent another key limitation in understanding diversification drivers in Elasmobranchii. Among palaeontological studies of diversification in elasmobranchs, the taxonomic scope of analyses is typically broad, often considering higher taxa such as Elasmobranchii (Guinot and Cavin, 2016; Kriwet and Benton, 2004; Kriwet and Klug, 2008; Kriwet et al., 2009; Underwood, 2006) or Chondrichthyes as a whole (Friedman and Sallan, 2012; Maisey et al., 2004; Sallan and Coates, 2010; Sallan and Galimberti, 2015). However, studies directly addressing drivers of diversification typically have a much narrower taxonomic focus (Boyd and Seitz, 2021; Brée et al., 2022; Claes et al., 2015; Condamine et al., 2019). Moreover, fossil diversity exceeds that observed today, but most extinct elasmobranchs are known only from isolated teeth (Cappetta, 2012), rendering species identification challenging. Among these studies, taxonomic level, taxon sampling and phylogenetic data vary substantially between studies (Table 1), making direct comparisons challenging.

Another confounding issue is the choice of variables that are investigated. For example, both Marion et al. (2024) and Mull et al. (2024) investigated relationships between diversification and body size (reaching contrasting conclusions), however interpretation of these results is clouded by the use of different body size measures (categorically binned and continuous variables respectively) and different phylogenies. Even if both studies included an identical list of taxa, these inconsistencies mean that there is no guarantee that qualitatively similar results would be produced. Future research should aim for methodological consistency, integrating fossil and neontological data where possible, and standardizing trait measures to enable direct comparisons across studies.

## 8 Conclusions

The underlying drivers of elasmobranch diversification dynamics remain poorly understood, although the case for specific biological traits, interactions, and environmental factors has been argued in recent studies (Table 1). When viewed holistically in the context of all available data, it is clear that all of these factors play critical roles in speciation and extinction, and that it is the interplay between them that drives diversification dynamics. Nevertheless, there is a clear absence of standardized, quantitative analyses considering alternative drivers of diversification in the literature. The potential influence of taxonomic level, phylogenetic uncertainty, and other limitations is rarely considered. Hence, future studies, considering both a greater range of biological traits and interactions, and environmental factors, may shed greater light onto diversification trends in elasmobranchs, and how these trends may have influenced the broader marine community.

## Figures and Tables

**Table 1:** Studies that have empirically tested for relationships between species diversification and biotic or abiotic covariates in elasmobranchs.

Study	Taxonomic coverage	Covariate(s)	Methodology
Claes et al., 2015	Assorted *Etmopterus*	Ventral and lateral luminescence	Comparative phylogenetic methods (MEDUSA)
Condamine et al., 2019	Assorted Lamniformes (350)	Tooth size, continental fragmentation index, global eustatic sea level, global temperature	Comparative phylogenetic methods (PyRate and BDCS)
Lamarca et al., 2024	Assorted Chondrichthyes (80)	Female sperm storage, multiple paternity	Comparative phylogenetic methods (MEDUSA)
Marion et al., 2024	Assorted Selachii (545)	Maximum body length, reproductive mode, ‘habitat’, diet	Comparative phylogenetic methods (SecSSE)
Mull et al., 2024	Assorted Elasmobranchii (610)	Reproductive mode, maximum body size, depth range, latitudinal range	Comparative phylogenetic methods (MEDUSA and MuSSE)

This does not include studies that have speculated, assumed, or inferred some relationship between trait evolution and diversification without empirical analysis finding a direct, statistically significant relationship between some biotic/abiotic variable and some measure of diversification rate. A comprehensive analysis of methodologies utilised by these studies is beyond the scope of this review; please see the respective studies for additional methodological details.
